# Quantitative Evaluation of Soil Structure and Strain in Three Dimensions under Shear Using X-ray Computed Tomography Image Analysis

**DOI:** 10.3390/jimaging7110230

**Published:** 2021-10-29

**Authors:** Shintaro Nohara, Toshifumi Mukunoki

**Affiliations:** 1 Central Research Institute of Electric Power Industry, 1646 Abiko, Abiko-shi 270-1194, Chiba, Japan; 2 Graduate School of Science and Technology, Kumamoto University, 2-39-1 Kurokami, Chuo-ku, Kumamoto-shi 860-8555, Kumamoto, Japan; 3 Faculty of Advanced Science and Technology, Kumamoto University, 2-39-1 Kurokami, Chuo-ku, Kumamoto-shi 860-8555, Kumamoto, Japan; mukunoki@kumamoto-u.ac.jp

**Keywords:** X-ray CT, soil fabric analysis, digital image correlation, strain localization, shear, soil structure

## Abstract

The objective of this study is to quantitatively evaluate the soil structure behavior when under shear stress to understand the mechanism of shear zone formation using a micro-focus X-ray computed tomography (CT) scanner to visualize the internal samples without causing disturbance. A new image-analysis method was proposed to systematically evaluate the particle length and direction by fitting the particle as an ellipsoid. Subsequently, a direct shear experiment was conducted on soil materials, and shear band was scanned using a micro-focus X-ray CT scanner. After validating the proposed method, the soil structure was evaluated in the shear zone via image analysis on the CT images. Furthermore, the strain inside the specimen was evaluated using digital image correlation. The results showed that a partial change in the particle direction occurred when the volume expansion inside the shear zone exceeded the peak. In addition, the width of the shear zone was ~7.1 times the median grain size of the sand used; however, the region exhibiting a change in the direction of the particles was narrow and confined to the vicinity of the shear plane.

## 1. Introduction

When a granular material is loaded, strains are localized within the material and eventually form a shear zone that leads to failure. It is well known that slippage between particles and rotation of particles have a significant effect on strain localization [1,2]. It has been found that the shear failure of granular materials is highly heterogeneous and not all the particles composing the granular material support the load [3,4,5]. Therefore, it is important to evaluate the behavior of sand using a microscopic approach. Furthermore, it is necessary to thoroughly understand the change of particle structure and the contact relationship between particles due to shearing, as well as construct a model that can reproduce the stress–strain relationship.

By quantitatively evaluating the microstructure of the actual granular material under deformation, a model may be created that corresponds to the actual behavior. However, compared to numerical approaches, there are not necessarily many examples of experimental investigations. In previous studies, detailed microscopic observations were made on thin specimens near the shear zone [6,7,8] and images of loaded specimens were evaluated [9]. Those studies attempted to evaluate the relationship between the shear zone formation and strain localization. However, the deformation behavior of granular materials is strongly influenced by the contact between the particles. Therefore, it is necessary to evaluate the particle structures in three dimensions in order to create a model that represents the actual behavior.

To overcome these difficulties, the effectiveness of X-ray computed tomography (CT) has recently been reported as a non-destructive method to evaluate the internal structure of sand [10,11,12,13]. In addition, micro-focus X-ray CT with a focal spot size of <50 μm has been developed for practical use, enabling visualization of soil particles as small as a few hundred micrometers [14,15,16]. Therefore, the changes of soil structure due to shearing can be evaluated in the same sample by utilizing X-ray CT. Furthermore, image analysis can quantitatively and objectively evaluate complex soil structures since CT images are digital data. Recent studies have attempted to quantitatively evaluate the contact between particles under the shear by CT image analysis [17,18,19,20]. Furthermore, it is also possible to quantitatively evaluate the distribution of strain from images by using analytical methods such as digital image correlation (DIC) [21,22,23]. From these backgrounds, the purpose of this study is to quantitatively clarify the effect of three-dimensional (3D) changes in particle structure due to shearing on strain localization by CT image analysis.

First, an image analysis method in which the particle fit to an ellipsoid is proposed to evaluate the shape and the direction of the particle in 3D space. The particle length and direction of the long, middle, and short axes can be evaluated simultaneously by fitting the particle as an ellipsoid. In addition, it has been reported that ellipsoidal particles can improve the numerical simulation results in a distinct element method (DEM) for granular materials since ellipsoidal particles are closer to the geometric shape of soil particles than spherical particles [24,25,26]. Therefore, the development of an ellipsoid-fitting method for soil particles with complex shapes, such as simple ellipsoids, will be useful for numerical calculations using ellipsoidal particles. In previous studies, the particle length and the direction of soil particles have been evaluated on CT images by direct measurement methods, such as using calipers [27,28], or principal component analysis [17,18,29]. However, these methods have not been evaluated based on the strict definition of the ellipsoid. Hence, it is necessary to develop a systematic image-analysis method that can fit not only particles that show simple shapes such as spheres or ellipsoids but also soil particles with complex shapes without changing the calculation algorithm. In this study, the effectiveness of a proposed method is verified by evaluating glass beads, resin particles manufactured by a 3D printer, and natural soil particles.

Next, to evaluate the influence region of structural changes due to shear deformation, CT imaging is performed on the specimens of direct shear experiment where the shear plane can strictly be made. Analysis for the CT images of the specimen at each shear displacement was conducted to quantitatively evaluate the soil structure changes due to shearing. Furthermore, DIC analysis is performed to evaluate the strain caused by shear. Finally, the effect of changes in soil structure on strain is investigated.

## 2. Materials and Method

### 2.1. Ellipsoid Fitting Method

An ellipsoid fitting method is proposed to evaluate the 3D shape and orientation of particles on CT images. An overview of the ellipsoid fitting method is presented in this section.

The general equation of an ellipsoid centered at the origin can be expressed as follows [30,31]:

(1) $${\mathrm{Ax}}^{2}+{\mathrm{By}}^{2}+{\mathrm{Cz}}^{2}+2\mathrm{Fyz}+2\mathrm{Gzx}+2\mathrm{Hxy}+D=0.$$

**Figure 1:**
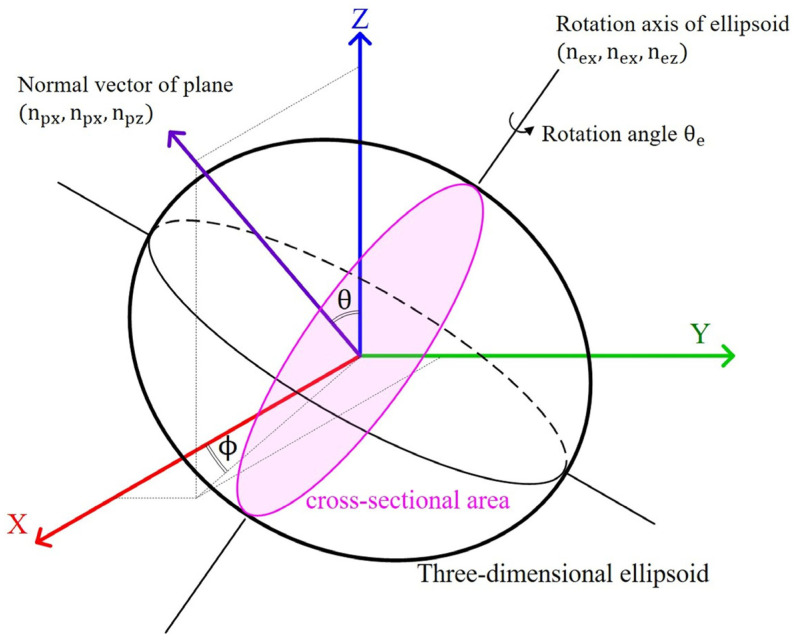
Schematic illustration of a three-dimensional ellipsoid with its center of gravity located at the origin.

There are seven indefinite parameters (A, B, C, D, F, G, and H). Here, the objective function is firstly defined about cross-sectional areas based on the particles extracted from CT images and the ellipsoid. Next, the objective function is minimized, using both Gauss–Newton method and by determining indefinite parameters of Equation (1), to minimize the error in the cross-sectional areas between the particle extracted from the CT image and the ellipsoid. Figure 1 shows a schematic illustration of the cross-sectional area of an ellipsoid.

If the direction vector starting at the origin is defined as Equation (2), the cross-sectional area of an ellipsoid with Equation (2) as its normal vector is expressed by Equation (3) [32,33,34].

(2) $$\begin{array}{cc} & n_{\mathrm{px}}x+n_{\mathrm{py}}y+n_{\mathrm{pz}}z=0,\\ & n_{\mathrm{px}}=\sin\theta \cos\phi ,\\ \mathrm{where}, & n_{\mathrm{py}}=\sin\theta \sin\phi ,\\ & n_{\mathrm{pz}}=\cos\theta . \end{array}$$

(3) $$\begin{array}{cc} & S_{e}=\frac{-D\pi }{\sqrt{e_{1}+e_{2}+e_{3}+e_{4}+e_{5}+e_{6}}}=\frac{-D\pi }{\sqrt{e}},\\ \mathrm{where}, & e_{1}=\left(\mathrm{BC}-F^{2}\right)n_{\mathrm{px}}^{2},\\ & e_{2}=\left(\mathrm{AC}-G^{2}\right)n_{\mathrm{py}}^{2},\\ & e_{3}=\left(\mathrm{AB}-H^{2}\right)n_{\mathrm{pz}}^{2},\\ & e_{4}=2\left(\mathrm{FG}-\mathrm{CH}\right)n_{\mathrm{px}}n_{\mathrm{py}},\\ & e_{5}=-2\left(\mathrm{BG}-\mathrm{FH}\right)n_{\mathrm{px}}n_{\mathrm{pz}},\\ & e_{6}=-2\left(\mathrm{AF}-\mathrm{GH}\right)n_{\mathrm{py}}n_{\mathrm{pz}}. \end{array}$$

where θ and ϕ are the angles defined for the normal vector (Figure 1). The derivation process of Equation (3) is shown in Appendix A.

If the cross-sectional area of a particle is calculated on the CT image as $S_{p}$, the objective function is defined as shown Equation (4).

(4) $$f_{\mathrm{obj}}=S_{p}-S_{e}=S_{p}-\frac{-D\pi }{\sqrt{e}}.$$

**Figure 2:**
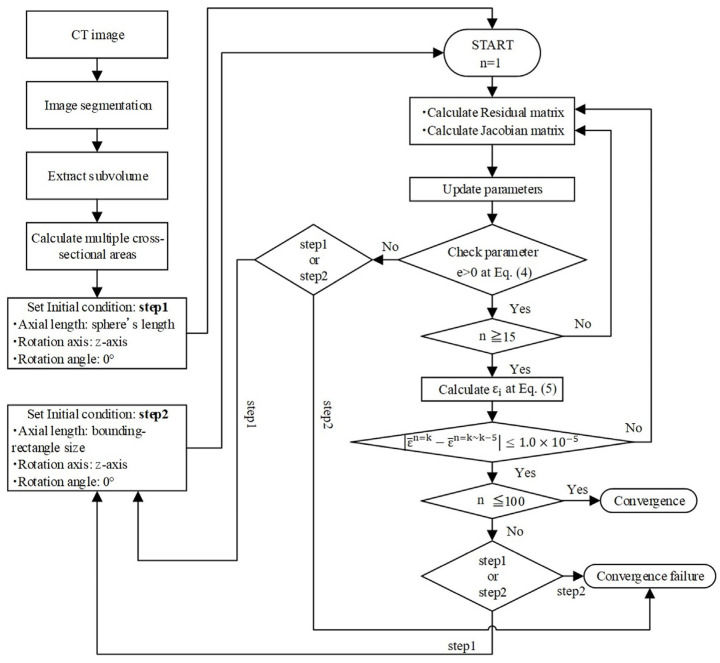
Ellipsoid fitting procedure using the Gauss–Newton method.

Figure 2 shows the evaluation procedure. First, the cross-sectional areas of a particle are calculated on segmented images. In this study, the cross-sectional areas are calculated at increments of 15° for θ and ϕ (0 ≤ θ, ϕ ≤ 180°). In total, 132 cross-sectional areas were calculated for each particle. Next, the initial values for the particle length are set to the spherical equivalent diameter, the rotation axis of the Z-axis, and the rotation angle of 0°, and the parameters are updated using the Gauss–Newton method. Then, the error of the cross-sectional area ($\varepsilon_{i}$ as shown in Equation (5)) between the particle and the ellipsoid is calculated. The convergence of iteration is judged by calculating the average of $\varepsilon_{i}$ for a particle, i.e., the iterative loop was broken if the difference was less than 10^−5^ between ${\bar{\varepsilon }}^{n=k}$ (which is the average of $\varepsilon_{i}$ at the present iteration) and ${\bar{\varepsilon }}^{n=k\sim k-5}$ (which is the average of $\varepsilon_{i}$ for the five iterations prior to the present iteration).

(5) $$\varepsilon_{i}=1.0-S_{p,i}/S_{e,i}\;\mathrm{if}\;S_{p,i}<S_{e,i}\;\varepsilon_{i}=1.0-S_{e,i}/S_{p,i}\;\mathrm{if}\;S_{p,i}\geq S_{e,i\;,}$$

where i = 1–m and m is the number of cross-sectional areas per particle. As described above, m was set to 132 in this study.

The minimum and maximum iterations of updates to the parameters for the Gauss–Newton method were set to 15 and 100, respectively. If the iterations do not converge after exceeding the maximum iterations, the initial values for the particle lengths are substituted using the values of the X-, Y-, Z-axis estimated by a bounding box, which is a rectangular bounding box around the particle. If the iterations still did not converge, ellipsoid fitting is discontinued. A calculation method for the particle length $d_{x}$, $d_{y}$, and $d_{z}$, with rotation axes $n_{\mathrm{ex}}$, $n_{\mathrm{ey}}$, and $n_{\mathrm{ez}}$, and rotation angle $\theta_{e}$, based on Equation (1), is shown in Appendix B.

In this study, the particles with a length of less than 10 voxels with respect to the X-, Y-, and Z-axes (measured using a bounding box) were not subjected to the ellipsoid fitting. This is because preliminary investigations have shown that if the number of voxels representing the particles is not sufficient, the cross-sectional area cannot be calculated accurately and in turn decreasing the accuracy of the ellipsoid fitting.

In order to fit the particle to an ellipsoid, image segmentation was conducted on CT images using the marker-controlled watershed method. The image segmentation presented here used the Python 3.4 open-source language programming software, with the NumPy, SciPy, and scikit-image libraries [35,36,37].

### 2.2. Materials

**Figure 3:**
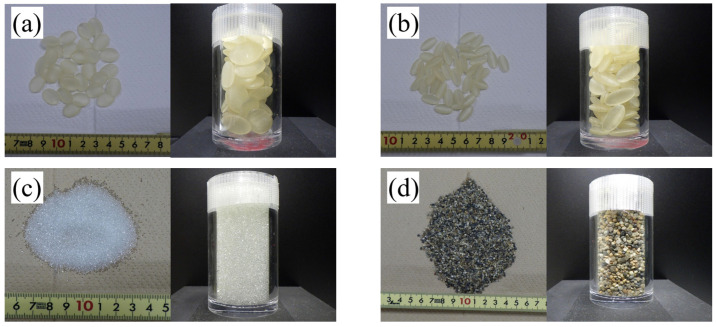
Acrylic glass columns filled with the materials: (**a**) RP1 and (**b**) RP2 (resin particles made by a 3D printer), (**c**) GBs (glass beads), and (**d**) KS (Kashima–Keisa natural sand).

In order to validate the ellipsoid fitting method proposed in this study, it is necessary to verify the accuracy of the proposed method by evaluation of not only natural soil particles but also particles with known shapes. Hence, four types of materials (resin particles: RP1, RP2; glass beads: GBs; Kashima–Keisa natural sand: KS) were scanned using a micro-focus X-ray CT scanner, and image analysis was conducted to evaluate the 3D characteristics of the particle shape. RP1 and RP2 are ellipsoid-shaped resin particles made by a 3D printer (Agilista-3200; Keyence, Osaka, Japan). For RP1, the particle lengths of the long-axis $d_{1}$, middle-axis $d_{2}$, and short-axis $d_{3}$ are 16, 12, and 4 mm, respectively. For RP2, $d_{1}$, $d_{2}$, and $d_{3}$ are 16, 8, and 4 mm, respectively. The density of RP1 and RP2 is 1060 kg/m^3^. GBs are commercially available glass beads with a smooth surface and an almost spherical shape. The particle size of a GB is 0.71–1.00 mm, and the true density is 2500 kg/m^3^. KS is a natural dry sand with a rough surface used to indicate angularity. The density of KS is 2640 kg/m^3^ and the particle size distribution is 1–3 mm. The photographs of the samples used in this study are shown in Figure 3.

### 2.3. Validation of the Ellipsoid Fitting Method

#### 2.3.1. CT Imaging and Particle Shape Evaluation

Four types of materials were filled into each acrylic glass column on a horizontal desk. The materials were not compacted, and the acrylic glass column had an internal diameter of 30 mm and a height of 60 mm, as shown in Figure 3. After CT scanning of the acrylic glass column using a micro-focus X-ray CT scanner (TXS-CT 450/160; Tesco, Yokohama, Japan), which was installed at the Central Research Institute of the Electric Power Industry in Japan, image segmentation was conducted. The TXS-CT 450/160 was equipped with two kinds of X-ray sources (450 kV and 160 kV sources). In this CT scanning, 160 kV source (maximum voltage is 160 kV) was used. Next, $d_{1}$, $d_{2}$, and $d_{3}$ were evaluated for all particles using the proposed ellipsoid fitting method. Finally, the shape parameters of $d_{3}/d_{1}$, $d_{2}/d_{1}$, $d_{3}/d_{2}$, and $S_{k}$ were calculated for each particle, where $d_{3}/d_{1}$, $d_{2}/d_{1}$, and $d_{3}/d_{2}$ indicate the aspect ratio, slenderness ratio, and flatness ratio, respectively, and $S_{k}$ indicates Krumbein’s sphericity [38] calculated using Equation (6).

(6) $$S_{k}=\sqrt[{3}]{\frac{d_{1}\cdot d_{2}\cdot d_{3}}{d_{1}^{3}}}.$$

The statistics, i.e., the arithmetic mean (Mean), standard deviation (Std), the first quartile value (Q_25_, 25%), the second quartile value (Q_50_, 50%), and the third quartile value (Q_75_, 75%), were calculated for $d_{1}$, $d_{2}$, $d_{3}$, and $S_{k}$. Furthermore, the evaluation accuracy for KS and the GBs were validated to obtain the cumulative curve for $d_{1}$, $d_{2}$, and $d_{3}$; histogram of $d_{3}/d_{1}$, $d_{2}/d_{1}$, and $d_{3}/d_{2}$; and histogram contour plot of a Zingg diagram [39].

#### 2.3.2. Particle Measurements Using a Particle Image Analyzer

A particle image analyzer (FF-30 micro; Jasco International, Tokyo, Japan, referred to as PIA) obtained 2D projection images of the particles using a high-performance digital camera while the sand particles were in free-fall and measured the shape characteristics of individual sand particles based on image analysis methods. The measuring range of PIA was 0.03 to 30 mm. If the particles are made of glass or resin, the measurement light will penetrate the particles, and the measurement will not be accurate. Hence, materials such as RP1, RP2, and GBs are not suitable for measurement using the PIA. Therefore, only the particle shape of KS was measured using the PIA. The PIA measured $d_{m}$, $e_{w}$, and $e_{l}$ for each soil particle, where $d_{m}$ is the area diameter, $e_{w}$ the width, and $e_{l}$ the length of the soil particle fit to the 2D ellipse ($e_{w}>e_{l}$).

A statistical analysis was performed on $d_{m}$ as well as $d_{1}$, $d_{2}$, and $d_{3}$, which are obtained by evaluating the CT images before comparing the results. As for $e_{w}/e_{l}$, the histograms are calculated as well as $d_{3}/d_{1}$, $d_{2}/d_{1}$, and $d_{3}/d_{2}$, which were obtained by evaluating the CT images before comparing the results.

### 2.4. Direct Shear Experiment with CT Imaging and Image Analysis

#### 2.4.1. Direct Shear Experiment

The direct shear experiment with a constant pressure was conducted using KS (Figure 3d). The specimen was scanned using a micro-focus X-ray CT scanner (TOSCANER-32300FPD, Toshiba IT & Control Systems, Tokyo, Japan), installed at Kumamoto University in Japan, to visualize soil structure after a certain amount of shear displacement. The maximum voltage of the TOSCANER-32300FPD was 230 kV. The material of the box used for the direct shear experiment was aluminum, considering X-ray penetration. The box had an internal diameter of 80 mm and height of 40 mm. The void ratio was 0.64. The specimens were divided into five layers and filled into the box. Vertical pressure was applied by a cylinder, and the vertical pressure during the test was 120 kPa.

The experiment apparatus used in this study was developed to perform the direct shear experiments in the X-ray CT shield room. Since a series of direct shear experiments were performed in an X-ray CT shield room, only stress released during the CT scan could be issued. Detailed specifications of the direct shear experiment apparatus used can be found in Chevalier et al., 2019 [40]. In this test apparatus, the upper shear box was fixed to a pillar and shear displacement was performed horizontally by manually rotating a screw attached to the lower shear box. The shear rate was set at 0.80 mm/min, and the shear was finished when the shear displacement reached 8.0 mm. In addition, the initial sheer condition was set to S0 and when the shear displacement reached 0.3, 3, 5, and 8 mm (S1 to S4), the shear displacement was stopped, and the entire shear box was scanned using micro-focus X-ray CT.

The watershed method was employed for image segmentation and particle extraction from the CT images. Then, image analysis of the CT images was used to quantitatively evaluate the change of soil structure with the progress of shear. In addition, DIC analysis was performed to determine the volumetric strain distribution and shear strain distribution inside the granular material as the shear progresses.

#### 2.4.2. Examination of Representative Volume Elements

On the CT images of the direct shear experiment 3D grids were created and the evaluation of changes in voids and interlocking between particles was conducted in each grid. To accomplish this, the size of the representative volume element (RVE) had to first be determined. Therefore, for the image of S0 of the direct shear experiment, isotropic 3D grids were created every 10 voxels with grid sizes ranging from 10 to 100 voxels, and the porosity was calculated. The grid size was determined through calculating the histogram of the porosity.

A grid was defined on the segmented image of the direct shear experiment using a structural grid. The porosity and the contact–surface ratio (CSR) between particles were calculated for each grid. The porosity means the ratio of non-particle voxels to RVE. CSR means the ratio of the voxels corresponding to the contact surface between particles to the RVE. The image analysis library used in this study allowed the user to select the definition of the watershed line for image segmentation using the watershed method [37]. Then, two images, one with the watershed line defined and the other without the watershed line defined, were created, and voxels corresponding to the contact surface between particles were extracted by subtracting the binarized images of the two.

In granular materials, it is known that the force is transferred at the contact points between particles, and the number of contact points per particle is called the coordination number. The higher the coordination number, the denser the soil structure and the better the interlocking is between particles. For this study, CSR is calculated to represent the interlocking between particles in a simplified representation. Hence, a larger CSR means that the contact surface in the RVE is larger and the soil structure is denser and better interlocked. In contrast, a smaller CSR means that the contact surface in RVE is smaller, the void is larger, and the interlocking between particles is lower.

#### 2.4.3. Analysis of Strain Localization by the Digital Image Correlation Method

DIC analysis is an image analysis method that evaluates the displacement field over the full field of a material by comparing two images [21,41]. The displacement is calculated from the correlation of 3D subsets of the reference and deformed volumetric images. From the displacement evaluation, the strain distribution can be obtained according to the displacement. DIC analysis was performed on CT images of direct shear experiments to obtain the volume strain distributions and shear strain distributions in 3D space using Tomowarp, an analysis code that can evaluate the displacement and strain between 3D images [21]. When the calculation of the correlation, the interval of the nodal point was set to 10 voxels in the X-, Y-, and Z-directions.

#### 2.4.4. Evaluation of the Particle Direction

The proposed method was performed to fit the ellipsoid as well as evaluate the particle length and direction of the long-, middle-, and short-axes for each particle on the CT images of the direct shear experiment. After calculating the intersection points between the long-, middle-, and short-axes of the best-fit ellipsoid, and for the upper and lower hemispheres of the unit sphere centered on the center of gravity of the best-fit ellipsoid, the intersection points were projected onto the horizontal (XY) and vertical (XZ) planes, and the frequency was calculated every 10°. The results of the calculation are summarized as a circular histogram using a rose diagram. Furthermore, to examine the change in particle direction with time, the histogram was divided into four regions (337.5–22.5° and 157.5–202.5°; 22.5–67.5° and 202.5–247.5°; 67.5–112.5° and 247.5–292.5°; 112.5–157.5° and 292.5–337.5°), and the frequencies were integrated in each region at each step.

## 3. Results

### 3.1. Validation of the Ellipsoid Fitting Method

The particles were fitted as ellipsoids using the proposed method on the CT images of four materials (RP1, RP2, GBs, KS) in acrylic glass columns, and their shapes were evaluated. In this section, the results are described.

#### 3.1.1. CT Images and Segmented Images

**Figure 4:**
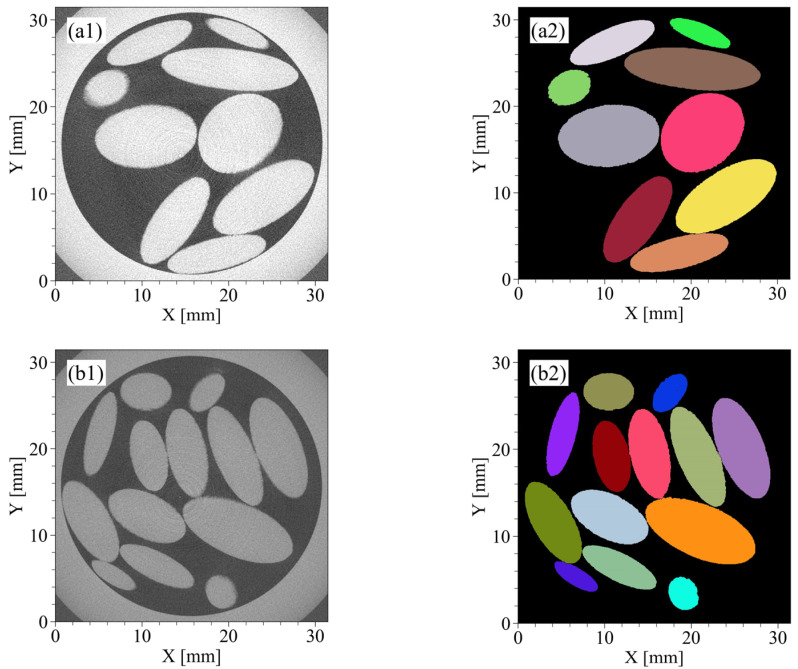

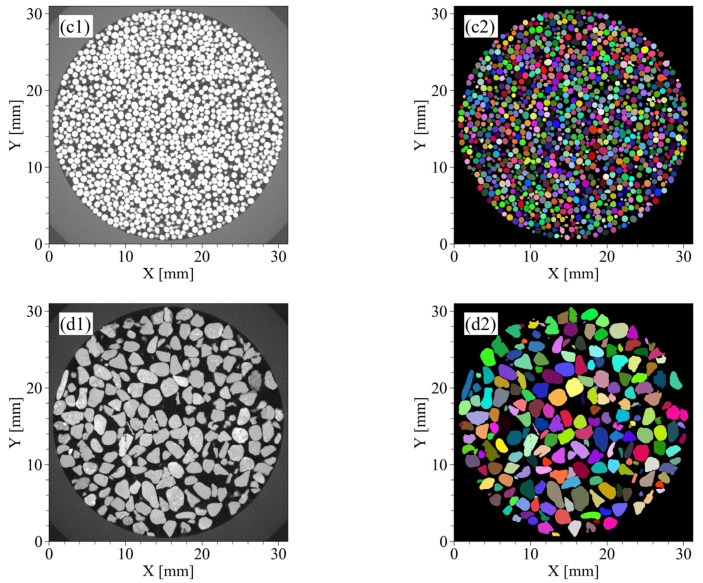
CT images and segmented images of four materials poured into acrylic glass columns: (**a1**,**a2**) RP1 and (**b1**,**b2**) RP2 (resin particles made by a 3D printer), (**c1**,**c2**) GBs (glass beads), and (**d1**,**d2**) KS (Kashima–Keisa natural sand). The left columns are CT images, and the right columns are segmented images.

Figure 4(a1–d1) shows CT images for the horizontal plane of four materials. The voxel size in Figure 4(a1,b1) is 45 μm/voxel and the image size is 930 × 930 × 1360 voxels. The voxel size in Figure 4(c1,d1) is 25 μm/voxels and the image size is 1560 × 1560 × 1360 voxels. The materials of RP1, RP2, and the GBs have smooth surfaces and simple shapes, such as spheres or ellipsoid, by direct observation of the CT images (Figure 4(a1–c1)). On the other hand, the materials of KS indicated angularity (Figure 4(d1)) with KS being natural sand.

Figure 4(a2–d2) shows segmented images for the horizontal plane of the four materials. In the case of RP1 and RP2 (Figure 4(a2,b2)), the segmentation was performed with good accuracy. In the case of GBs and KS (Figure 4(c2,d2)), there were some particles where either multiple particles were extracted as one particle or one particle was extracted as multiple particles but the segmentation was generally successfully performed. As a result of image segmentation, the number of particles was 46 for RP1; 76 for RP2; 47,634 for GBs; and 3381 for KS.

#### 3.1.2. Ellipsoid Fitting

When the size of the particle to be analyzed is too small to visualize the 3D image of soil particle properly, the voxels representing the particle are not sufficient to accurately calculate the cross-sectional area of the particle, thereby decreasing the ellipsoid fitting accuracy. Therefore, an ellipsoid fitting was performed for particles that had particle length greater than 10 voxels with respect to the X-, Y-, and Z-axes using a bounding box for evaluation. As a result, the total number of fitting ellipsoids obtained for each material were: 44 for RP1; 76 for RP2; 3100 for KS; and 46,899 for GBs. For RP1, there was a difference between the number of particles in the segmented image and the number of particles in the fitting result. This is due to the effect of over-segmentation during the segmentation process, and the particles that should have been one were partially identified as different particles.

**Figure 5:**
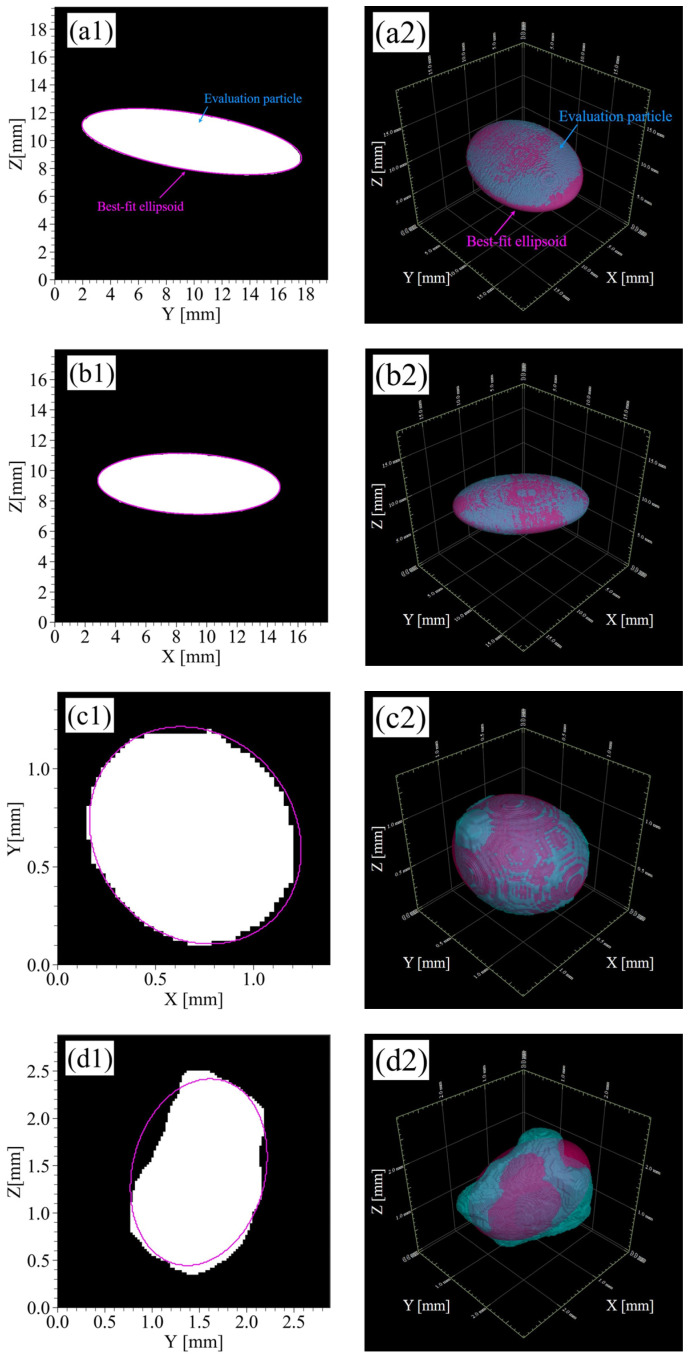
Ellipsoid fitting results: (**a1**,**a2**) RP1 and (**b1**,**b2**) RP2 (resin particles made by a 3D printer), (**c1**,**c2**) GBs (glass beads), and (**d1**,**d2**) KS (Kashima–Keisa natural sand). The left columns are the two-dimensional views, and the right columns are the three-dimensional views.

Figure 5 shows some fitting results for the four materials. In the case of RP1 and RP2, which were made using a 3D printer (Figure 5(a1,a2,b1,b2)), their ellipsoid approximation was achieved with good accuracy. In the case of the GBs, which are almost spheres, some particles have protrusions as shown in Figure 5(c1,c2). However, the proposed method was able to adequately approximate the ellipsoids without being affected by the protrusions. Although the shape of KS was more complicated (Figure 5(d1,d2)) than that of the GBs, RP1, and RP2, and some protrusions or were necks observed. However, the particles were reasonably fitted as ellipsoids.

#### 3.1.3. Evaluation of the Particle Shape Characteristics

**Figure 6:**
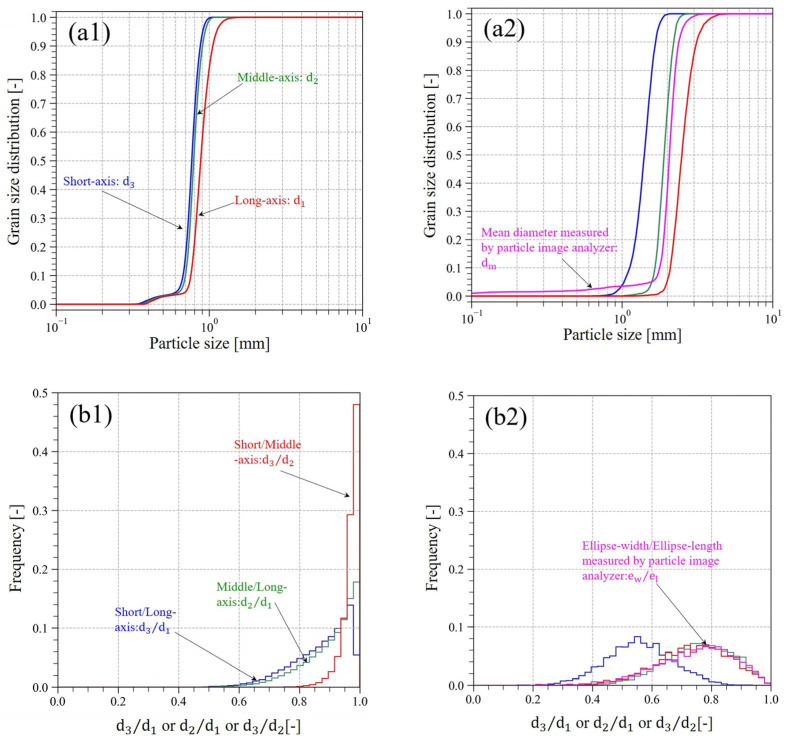

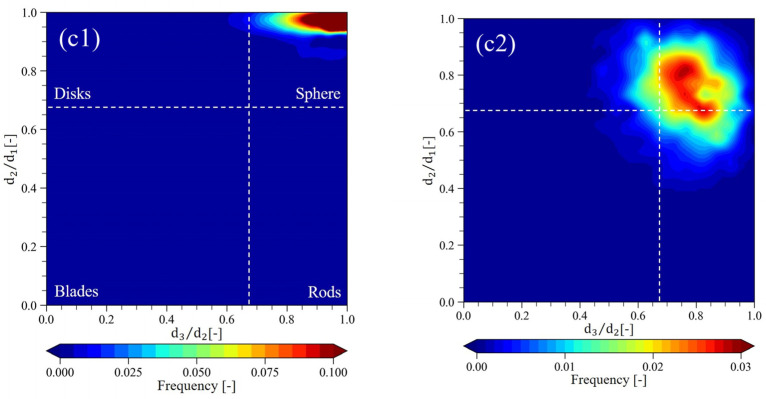
Evaluation results of particle shape. (**a1**,**a2**) are the cumulative ratio curve of $d_{1}$, $d_{2}$, $d_{3}$, and $d_{m}$ (only for KS). (**b1**,**b2**) are the histograms of $d_{3}/d_{1}$, $d_{2}/d_{1}$, $d_{3}/d_{2}$, and $e_{w}/e_{l}$ (only for KS). (**c1**,**c2**) are Zingg diagrams. The left columns are evaluation results of the GBs, and the right columns for KS. GBs, glass beads; KS, Kashima–Keisa natural sand.

Table 1 lists the statistical analysis results for $d_{1}$, $d_{2}$, $d_{3}$, and $S_{k}$ that were obtained by fitting ellipsoids. The statistical analysis of $d_{m}$ for KS measuring by PIA is also listed in Table 1. The cumulative curves for $d_{1}$, $d_{2}$, and $d_{3}$ of the GBs and KS are shown in Figure 6(a1,a2). The histograms for $d_{3}/d_{1}$, $d_{2}/d_{1}$, and $d_{3}/d_{2}$ of the GBs and KS are shown in Figure 6(b1,b2). The measuring results of $d_{m}$ and $e_{w}/e_{l}$ for KS are also shown in Figure 6(a2,b2). The histogram contour plots of the Zingg diagrams for KS and the GBs are shown in Figure 6(c1,c2).

As shown in Table 1, the Q_50_ of $d_{1}$, $d_{2}$, and $d_{3}$ for RP1 and RP2 were approximately equal to the manufacturing conditions, with a maximum error of 0.17 mm (4 voxels). Q_50_ of $d_{1}$, $d_{2}$, and $d_{3}$ for the GBs were within the range of 0.7–1.0 mm, which is the specification of the particle size of the glass beads. Furthermore, Q_50_ of $d_{1}$, $d_{2}$, and $d_{3}$ for KS evaluated from the CT images were not significantly different from Q_50_ of $d_{m}$ measured using PIA. Q_50_ of $S_{k}$ calculated by Equation (6) was 0.94 for the GBs since the glass beads represented a sphere and were expected to have a sphericity close to 1. Figure 4(c1) shows that some glass beads were not sphere, but more ellipsoid. This may have affected the sphericity, which was not exactly equal to 1.

As shown in Figure 6(a1), there was no difference in the particle size distributions of $d_{1}$, $d_{2}$, and $d_{3}$ in the GBs. This indicates that there was no significant difference between $d_{1}$, $d_{2}$, and $d_{3}$ because of the near-spherical shape of the GBs. On the other hand, Figure 6(a2) shows that the difference between the particle size distributions for $d_{1}$, $d_{2}$, and $d_{3}$, as well as $d_{m}$, when measured by PIA, occurred in the region where the curve rises. This was due to the minimum particle length of 0.03 mm of PIA, while particles with a diameter of 0.25 mm or more were used for evaluation on the CT images. In contrast, the gradients of the curves evaluated from the CT images and the gradients of the curves measured by PIA became generally equivalent as the particle length increased. The particle size distribution of $d_{m}$ was close to that of either $d_{1}$ or $d_{2}$, this may have been influenced by the measurement method of PIA. Since PIA captured the image during free fall, it has been reported that the particle length measured by PIA is strongly influenced by the direction in which the air resistance is reduced, i.e., the long axis of the particle [42]. Hence, the particle size distribution of $d_{m}$ is close to the relatively large particle size distribution.

As shown in Figure 6(b1), the peak of the histogram was concentrated at ~1 because the shape of the GBs were almost sphere. As described above, not only spherical but also ellipsoidal glass beads were observed on the CT images (Figure 4(c1)), which may have caused the difference in the shape of the histogram between $d_{3}/d_{1}$, $d_{2}/d_{1}$, and $d_{3}/d_{2}$. As shown in Figure 6(b2), the histograms of $e_{w}/e_{l}$ measured by PIA and the histograms of $d_{2}/d_{1}$ and $d_{3}/d_{2}$ evaluated from the CT images were in close correspondence.

Figure 6(c1,c2) shows the histogram contour plot with $d_{2}/d_{1}$ on the vertical axis and $d_{3}/d_{2}$ on the horizontal axis. In these figures, the value of $d_{2}/d_{1}$ and $d_{3}/d_{2}$ at 2/3 (≈0.67) is taken as the boundary value, and the particles are classified into spheres, rods, disks, and blades. As shown in Figure 6(c1), almost all particles in the GBs were classified as spheres. On the other hand, most of the particles in KS were also classified as spheres, but some particles were classified as disks or rods, with much more variation compared to the GBs (Figure 6(c2)). This indicates that KS is natural sand, composed of soil particles of various shapes.

$d_{1}$, $d_{2}$, and $d_{3}$ were evaluated from the CT images by ellipsoid fitting and $d_{m}$ was obtained by the particle image analyzer for KS. RP1 and RP2, resin particles (made by a 3D printer); GBs, glass beads; KS, Kashima–Keisa natural sand. For RP1, the actual particle lengths of $d_{1}$, $d_{2}$, and $d_{3}$ were 16, 12, and 4 mm, respectively. For RP2, the actual particle lengths of $d_{1}$, $d_{2}$, and $d_{3}$ were 16, 8, and 4 mm, respectively.

### 3.2. Direct Shear Experiment

#### 3.2.1. Experimental Results, CT Images, and Segmented Images

**Figure 7:**
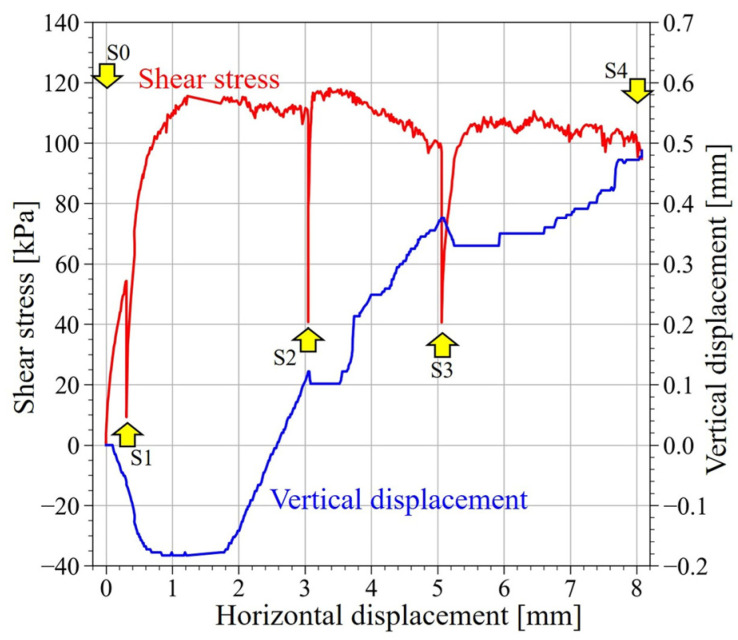
Experimental results of direct shear experiment. CT scanning was performed when horizontal displacement was 0.0 (S0), 0.3 (S1), 3.0 (S2), 5.0 (S3), and 8.0 mm (S4).

Figure 7 shows the temporal changes of shear stress and vertical displacement in the direct shear experiment. The shear stress peaked when the horizontal displacement (D_h_) reached ~1.3 mm. After the peak of shear stress, the shear stress gradually decreased, and the material shifted to the strain softening process. Until D_h_ reached 0.6 mm, the vertical displacement was decreasing and the whole specimen was shrinking in volume. When D_h_ reached 1.8 mm, the vertical displacement began to increase and the whole specimen expanded in volume. In particular, the vertical displacement increased rapidly from S1 (D_h_ = 0.3 mm) to S2 (D_h_ = 3 mm) and from S2 to S3 (D_h_ = 5 mm).

In this experiment, the shear process was interrupted because CT imaging was repeated when D_h_ reached a certain displacement. Hence, the specimen was considered to be affected by the shear stress relaxation to a certain degree, as shown in Figure 7. However, the vertical load was still applied during the CT imaging, and the affection on the internal structure was considered to be insignificant.

**Figure 8:**
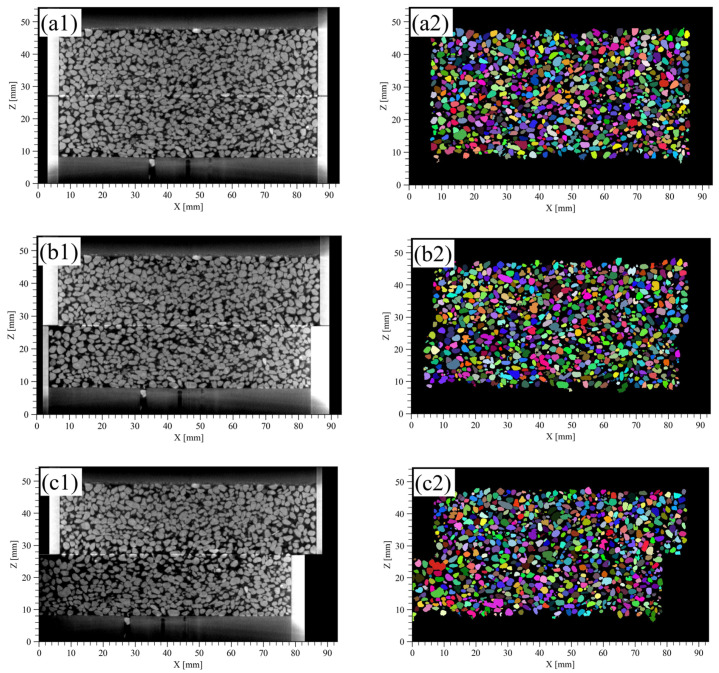
CT images and segmented images of the direct shear experiment on the vertical plane (XZ plane). (**a1**,**a2**) are the results at S0 (initial sate). (**b1**,**b2**) are the results at S2 (D_h_ = 3.0 mm). (**c1**,**c2**) are the results at S4 (D_h_ = 8.0 mm). D_h_, horizontal displacement. The left columns are CT images, and the right columns are segmented images. The boundary surface between the upper and lower boxes is at a position where the Z-axis is about 27 mm. The lower box is displaced horizontally from the +X to the −X direction.

Figure 8(a1–c1) shows the CT images at S0 (initial state), S2 (D_h_ = 3 mm), and S4 (D_h_ = 8 mm). The voxel size was 91 μm/voxel, and the image size was 1024 × 1024 × 600 voxels. In this experiment, the shear stress reached its peak when D_h_ reached ~1.3 mm, so the images after S2 are the CT images during the strain softening process. In S0 (Figure 8(a1)), the voids appeared to be homogeneous over the sample, but after S2 (Figure 8(b1,c1)), the voids near the shear plane increased.

Figure 8(a2–c2) show the segmented images at S0, S2, and S4 using the watershed method. There were some instances where multiple particles were extracted as one particle or one particle was extracted as multiple particles, but the segmentation was generally successfully performed. As a result of image segmentation, the number of particles was 29,233 for S0; 28,683 for S2; and 27,995 for S4. In S4, the number of particles decreased, which may be due to the effect created by part of the lower box outside the field of view that deformed the lower box horizontally.

#### 3.2.2. Evaluation of the Porosity and Contact Surface between Particles

**Figure 9:**
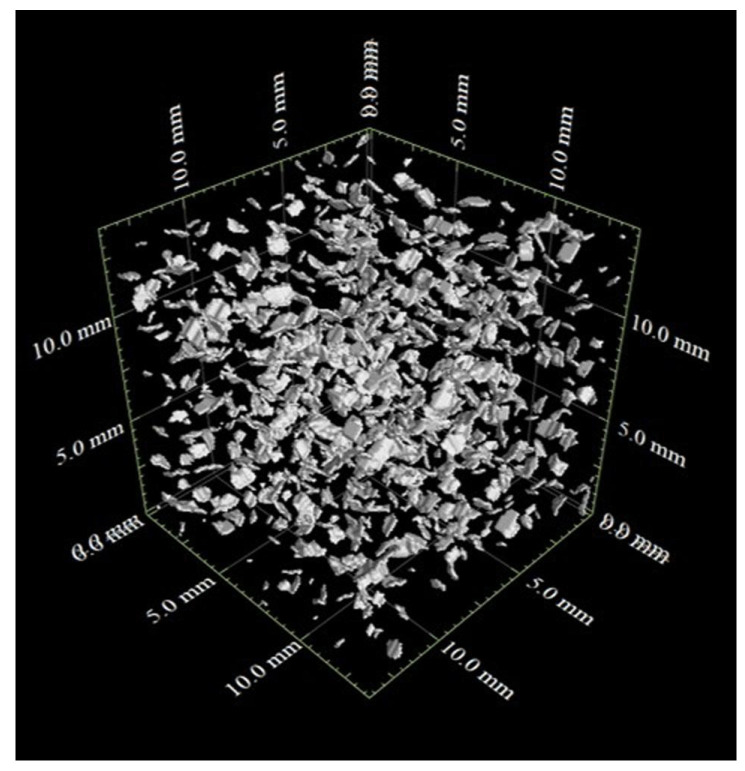
Contact surface between particles in three-dimensional view.

**Figure 10:**
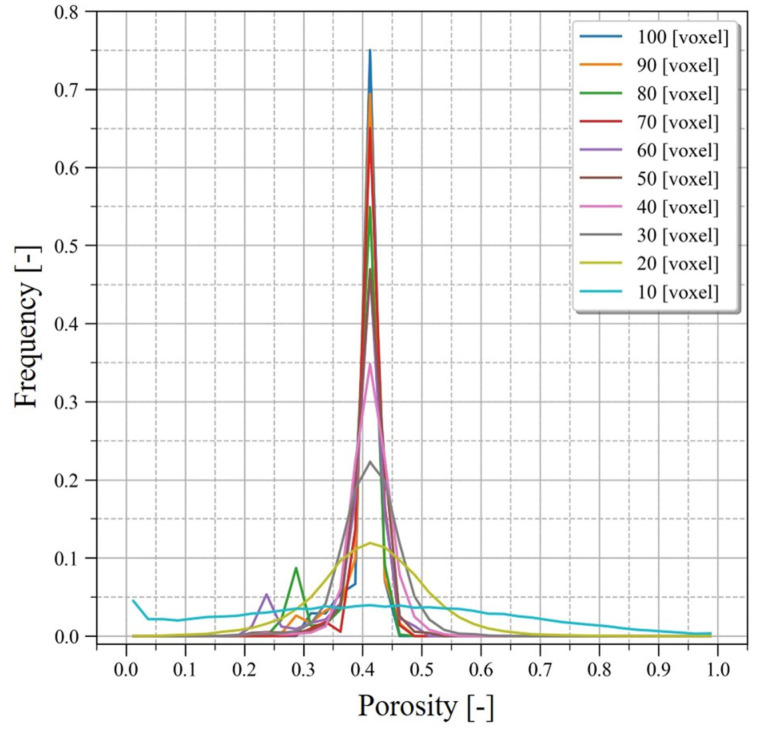
Histogram of the porosity. The porosity was calculated as the grid size of the representative volume element every 10 voxels between 10 and 100 voxels for S0.

Figure 9 indicates the watershed line in 3D space corresponding to the contact surface between particles extracted by the method describe above. The contact surface between the particles was confirmed to be flat or curved. Figure 10 shows the calculation results of the porosity in each grid by creating isotropic 3D grids with grid sizes from 10–100 voxels on the segmented image of S0 (Figure 8(a2)). The frequency of porosity in the histogram was calculated every 0.05 in the sand-filled region.

A grid size of 10 voxels was not adequate since some grids had zero porosity; i.e., only particles occupied the grid. When the grid size was larger than 10 voxels, the peak was observed when porosity was between 0.40 and 0.45. When the grid size exceeded 40 voxels, only the peaks became higher while the shape of the bottom part of the histogram remained approximately the same. This means that there was no change in the porosity distribution even when the grid size was further increased. Based on the results, the grid size was set to 40 voxels (3.64 mm) in consideration of the image resolution.

**Figure 11:**
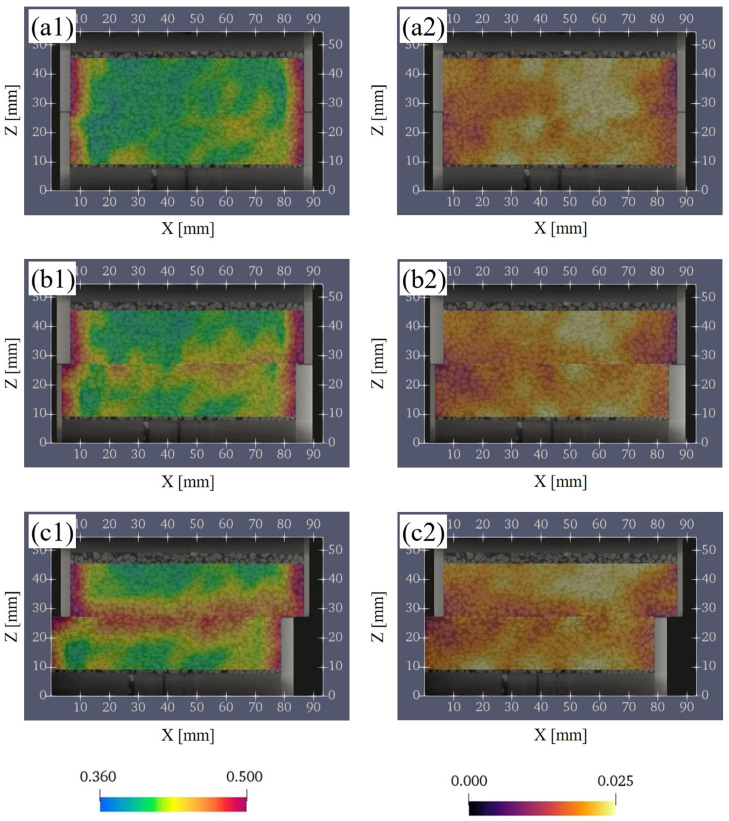
Evaluation results of the porosity and the ratio of contact-surface between particles on the vertical plane (XZ plane). (**a1**,**a2**) are the results at S0 (initial sate). (**b1**,**b2**) are the results at S2 (D_h_ = 3.0 mm). (**c1**,**c2**) are the results at S4 (D_h_ = 8.0 mm). D_h_, horizontal displacement. The left columns are the porosity, and the right columns are the ratio of contact-surface between particles.

Figure 11(a1–c1) shows the evaluation results of porosity for S0, S2, and S4. In S0 (Figure 11(a1)), the distribution of porosity was generally uniform except near the boundary between the sand and the vessel. However, as the shear progressed and shifted to strain softening process (Figure 11(b1)), the porosity near the shear plane increased locally. In S4 (Figure 11(c1)), the porosity near the shear plane increased uniformly, and there was a continuous high porosity in the horizontal direction.

Figure 11(a2–c2) shows the evaluation results of the CSR for S0, S2, and S4. CSR means the ratio of the voxels corresponding to the contact surface between particles the to RVE. The greater the CSR, the stronger the interlocking of the particles was. In S0 (Figure 11(a2)), the CSR was heterogeneously distributed, and there was a mixture of low and high CSR in the whole specimen. Then, as the shear progressed (Figure 11(b2,c2)), CSR near the shear plane decreased. However, CSR did not decrease as uniformly as porosity, and grids with locally high CSR were also observed near the shear plane.

#### 3.2.3. The Change of the Porosity and the Contact Surface between Particles

**Figure 12:**
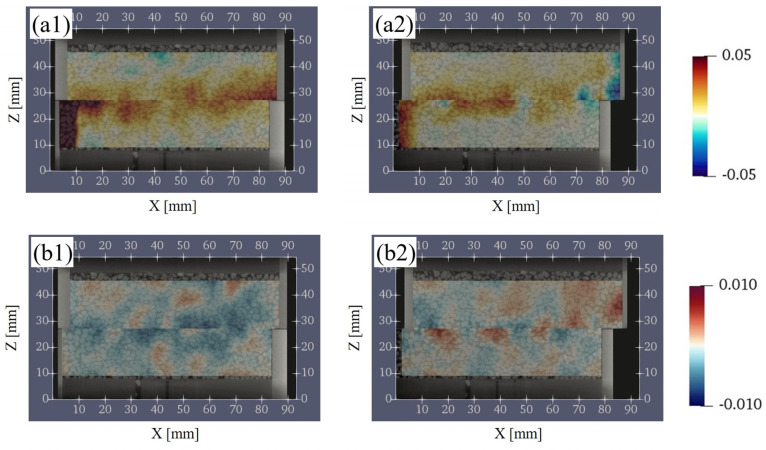
Change in the porosity and the ratio of contact surface between particles on the vertical plane (XZ plane). (**a1**,**a2**) show the change in the porosity. (**b1**,**b2**) show the change in the ratio of contact surface between particles. The left columns represent the change between S1 (D_h_ = 0.3 mm) and S2 (D_h_ = 3 mm), and the right columns represent the change between S3 (D_h_ = 5 mm) and S4 (D_h_ = 8 mm). D_h_, horizontal displacement.

In this section, the change of the porosity and CSR were calculated between before and after each shear displacement. Figure 12(a1,b1) shows the calculation results of subtracting S1 from S2 and Figure 12(a2,b2) shows the calculation results of subtracting S4 from S3 about the porosity and CSR. Both results were calculated by considering the displacement of the lower box and depended on the position of the box.

As the shear progressed from S1 (D_h_ = 0.3 mm) to S2 (D_h_ = 3 mm), the porosity increased over a wide region in the vertical direction around the shear plane (Figure 12(a1)). As for the CSR, the decreasing tendency near the shear plane was remarkable (Figure 12(b1)). This indicated that the contact area decreased due to the loss of interlocking between the particles as a result of shearing. In addition, the increase in the porosity and the decrease in the CSR generally correspond to each other near the shear plane.

As the shear progressed from S3 (D_h_ = 5 mm) to S4 (D_h_ = 8 mm), the porosity near the shear plane increased (Figure 12(a2)) similar to the change from S1 to S2. However, the region of increase in the porosity is smaller than the change from S1 to S2 and remained near the shear plane. The CSR was a mixture of increasing and decreasing regions near the shear plane (Figure 12(b2)). In addition, the increase in the porosity and the decrease in CSR generally did not necessarily correspond to each other near the shear plane.

#### 3.2.4. Evaluation of the Volumetric Strain and the Shear Strain

**Figure 13:**
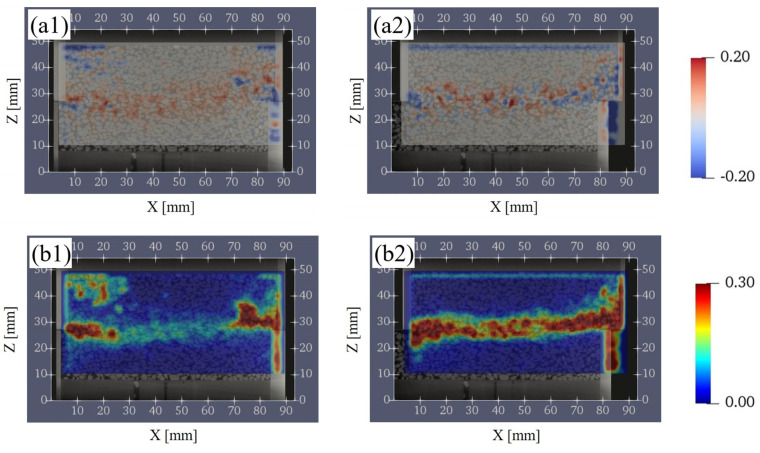
Digital image correlation (DIC) analysis results on the vertical plane (XZ plane). (**a1**,**a2**) are the volumetric strain, while (**b1**,**b2**) are the shear strain. The left columns show the analysis results based on the CT images of S1 (D_h_ = 0.3 mm) and S2 (D_h_ = 3 mm), and the right columns show the analysis results based on the CT images of S3 (D_h_ = 5 mm) and S4 (D_h_ = 8 mm). D_h_, horizontal displacement.

Figure 13(a1,a2) shows the distribution of volumetric strain, and Figure 13(b1,b2) shows the distribution of shear strain. Figure 13(a1,b1) shows the DIC analysis results from S1 to S2, and Figure 13(a2,b2) shows the DIC analysis results from S3 to S4.

As shown in Figure 13(a1), the volume expansion was dominant in the shear zone from S1 to S2. This result was also in agreement with the variation of the vertical displacement with time, shown in Figure 7. From S3 to S4, the volume shrinkage and the expansion were observed in the shear zone (Figure 13(a2)), indicating a complex destruction mode characteristic of granular materials. On the other hand, Figure 13(b1) shows that local shear strain occurred from S1 to S2. From S3 to S4 (Figure 13(b2)), no significant shear strain was observed except near the shear plane and a clear continuous horizontal shear zone was formed.

#### 3.2.5. Comparison of the Porosity, the Contact Surface Ratio, the Volumetric Strain, and the Shear Strain versus Distance from the Shear Plane

**Figure 14:**
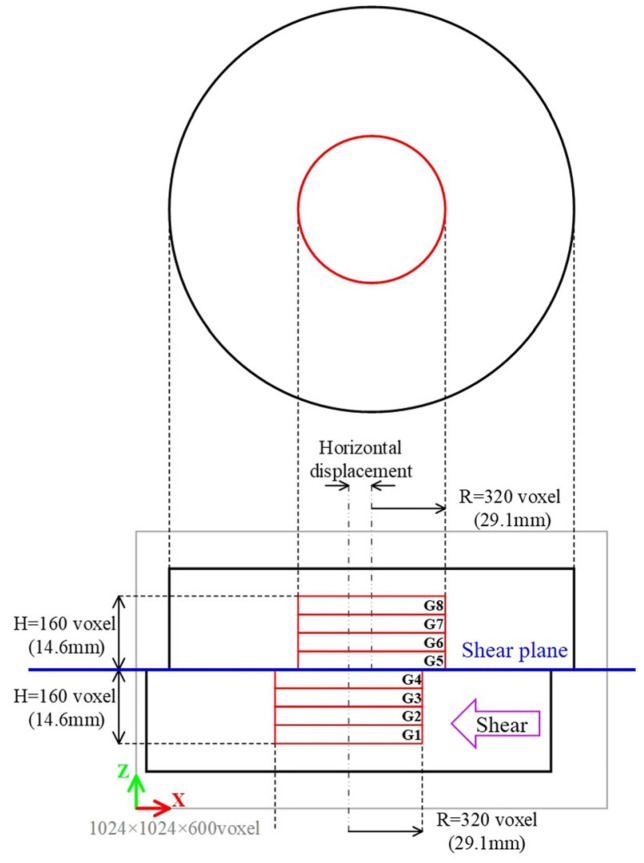
Grouping in the shear box according to the distance from the shear plane.

**Figure 15:**
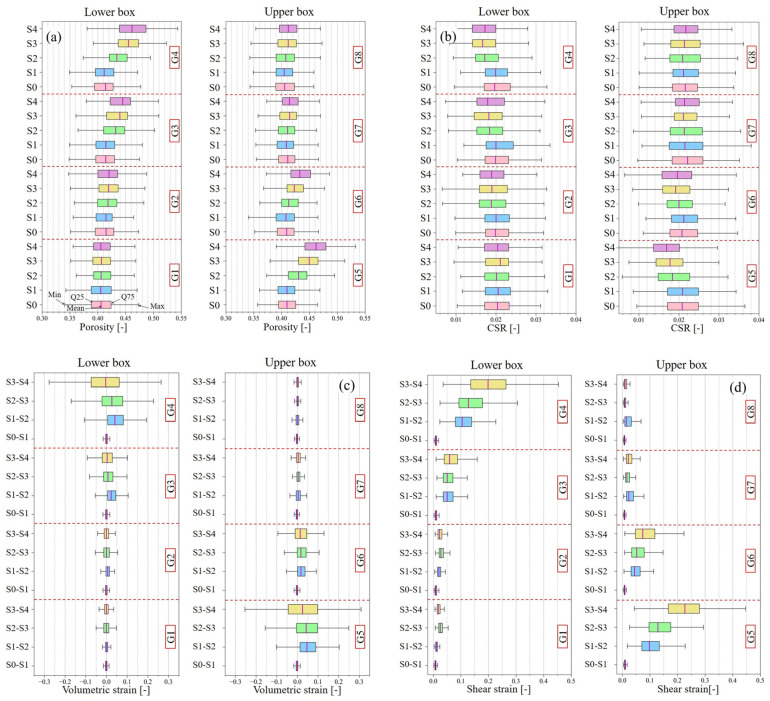
Statistical analysis (represented as box plots) for each group: (**a**) porosity, (**b**) ratio of contact surface between particles, (**c**) volumetric strain, and (**d**) shear strain.

As shown in Figure 14, the range of 320 voxels in the radial direction and 320 voxels in the height direction from the center of the box was divided into eight groups in the height direction. Then, statistical analysis was conducted for each group about the results of the porosity, CSR, volumetric strain, and shear strain, and the results are summarized as box plots (Figure 15). In both results, there was negligible change in G1–G2, and G7–G8, which are farther from the shear plane, and significant change occurred in the range of ~7 mm from the shear plane (G3–G4 and G5–G6).

As shown in Figure 15a, the porosity increased rapidly from S1 to S2 in the range of G3–G6 and the average of the porosity increased monotonously after S2. However, the change in porosity from S3 to S4 was relatively gradual, and the porosity inside the shear zone increased in variability. As for CSR, it decreased rapidly from S1 to S2 (Figure 15b). After S2, the decreasing tendency of the CSR became slower or remained almost unchanged, showing a different tendency from that of the porosity. As for the volume strain, the mean value of the volume strain was positive until S3, and the volume expansion was dominant (Figure 15c). After S3, the minimum value of the volumetric strain decreased and the maximum value of the volumetric strain increased, indicating that the variation was increasing near the shear plane. However, the average value of the volumetric strain was close to zero, indicating that the volumetric expansion and shrinkage were well balanced. As for the shear strain, it monotonically increased after S1 (Figure 15d).

#### 3.2.6. Evaluation for the Particle Direction

**Figure 16:**
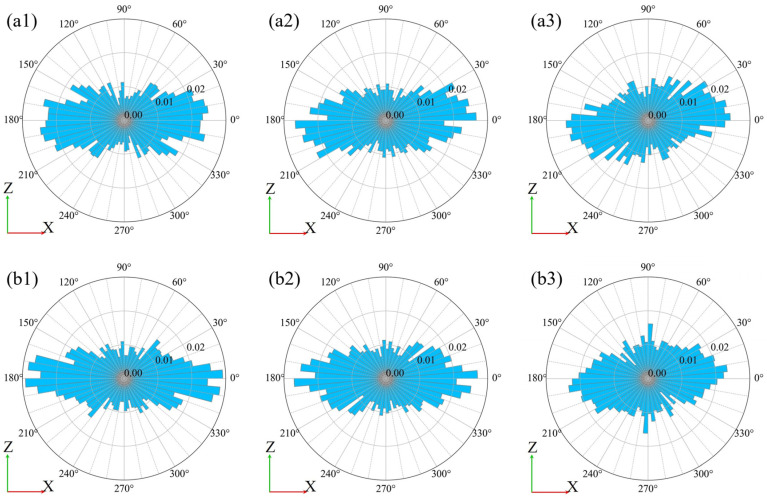
Histograms of particle direction of the long axis on the vertical plane (XZ plane): (**a1**–**a3**) are G5; (**b1**–**b3**) are G4. The left columns represent S0 (initial state), the center columns S2 (D_h_ = 3 mm), and the right columns S4 (D_h_ = 8 mm). D_h_, horizontal displacement.

**Figure 17:**
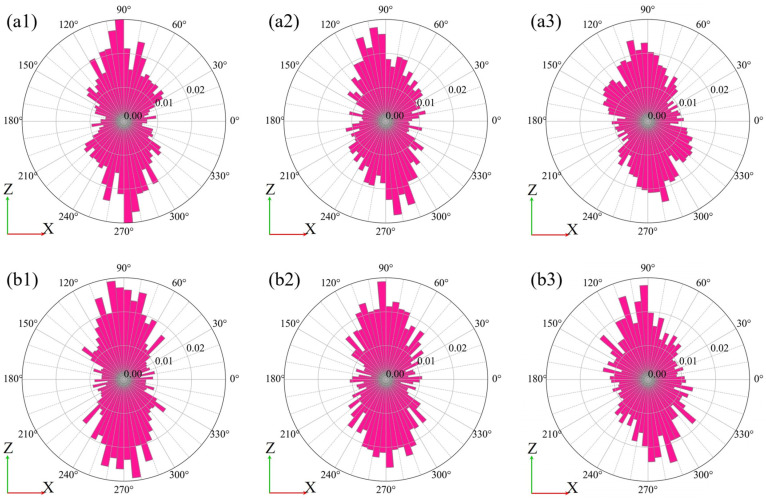
Histograms of particle direction of the short axis on the vertical plane (XZ plane): (**a1**–**a3**) are G5; (**b1**–**b3**) are G4. The left columns represent S0 (initial state), center columns represent S2 (D_h_ = 3 mm), and right columns represent S4 (D_h_ = 8 mm). D_h_, horizontal displacement.

Figure 16 and Figure 17 show the results of calculating the histograms for the particle directions based on the group classification shown in Figure 14. Figure 16 shows the direction of the particle’s long axis in the vertical (XZ) plane, Figure 17 shows the direction of the particle’s short axis in the vertical (XZ) plane, and the results for G3–G6 in S0, S2, and S4. The number of particles in G4 to G5 was 1280–1500.

Figure 16(a1,b1) shows that most of the long axes of the particles were directed horizontally (337.5–22.5° and 157.5–202.5°) in S0. In particular, the long axes of ~40% of the particles were directed horizontally in G4 and G5. Figure 17(a1,b1) shows that most of the short axes of the particles were directed vertically (67.5–112.5° and 247.5–292.5°) in S0. In particular, the short axes of ~43% of the particles were directed vertically in G4 and G5. As the shearing progressed to S2 (D_h_ = 3 mm), some particles were visually observed to rotate (Figure 8(b1,b2)), but the direction of most particles did not change significantly from S0. Even when the shear progressed to reach S4 (D_h_ = 8 mm), there was no significant change in the overall direction of the particles (Figure 8(c1,c2)).

**Figure 18:**
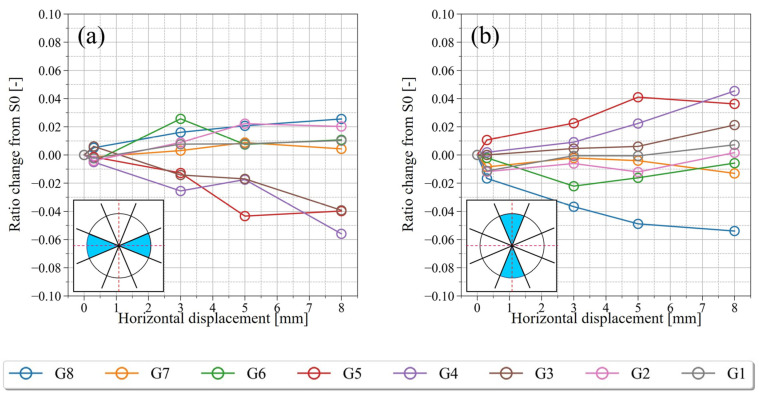
Variation in the ratio of the particle’s long axis from S0 (initial state). (**a**) 337.5–22.5° and 157.5–202.5°, and (**b**) 67.5–112.5° and 247.5–292.5°.

**Figure 19:**
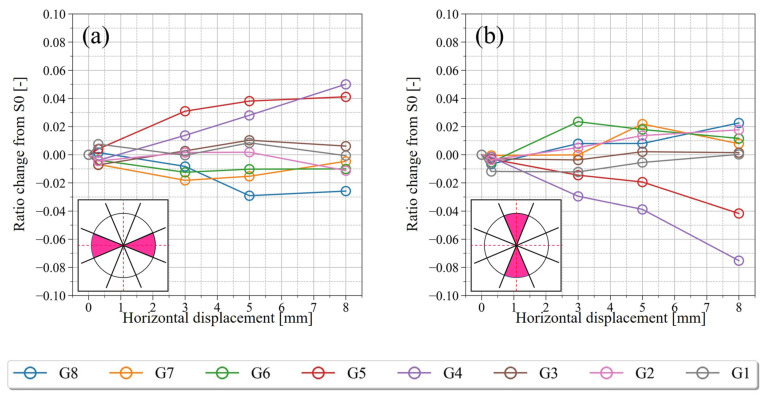
Variation in the ratio of the particle’s short axis from S0 (initial state). (**a**) 337.5–22.5° and 157.5–202.5°, and (**b**) 67.5–112.5° and 247.5–292.5°.

Figure 18 and Figure 19 shows the results of integrating the frequencies of a region of directions at each step: horizontal (337.5–22.5° and 157.5–202.5°) and vertical (67.5–112.5° and 247.5–292.5°). These parameters are classified according to the group classification (G1–G8) shown in Figure 14 and indicate the change in the direction of the particles over time. Figure 18 shows the results for the particles’ long axes and Figure 19 shows the results for the particles’ short axes, where the vertical axis represents the rate of change from S0, and the horizontal axis represents the magnitude of shear displacement.

According to the change in the direction of the particles with time (Figure 18 and Figure 19), a remarkable change in the direction of the particles occurred at G4 and G5, which were close to the shear plane. In both regions, the change was relatively gradual until S2 (D_h_ = 3 mm), but it appeared to have become increasingly rapid after S3. As shown in Figure 18a, which is the temporal change of the horizontal particle long axis, G4 and G5 showed a different tendency from G1–G3 and G6–G8, and the ratio of the horizontal long axes of the particles decreased monotonically, with a maximum decrease of ~6%. As shown in Figure 18b, which is the temporal change of the vertical particle long axis, the ratio of the vertical long axes of the particles increased monotonically in G4 and G5, with a maximum increase of ~4%. In the regions other than G4 and G5, except for G3, the ratio of the vertically directed long axes of the particles remained almost unchanged or showed a decreasing tendency. In the histogram for the particle long axis of G4 in S4 (Figure 16(b3)), peaks were also observed in the 90° and 270° directions. The increase in the ratio of the vertically directed long axes of the particles can be considered as one of the changes in soil structure due to shearing.

As shown in Figure 19a,b, the ratio of the horizontally directed short axes of the particles increased monotonically, and the ratio of the vertically directed short axes of the particles decreased monotonically in G4 and G5. In particular, a remarkable change was observed in G4, where the ratio of the vertically directed short axes of the particles decreased by a maximum of 8%. These changes are considered to be one of the changes in soil structure due to shearing, since they showed a different tendency from the particles in G1–G3 and G6–G8.

## 4. Discussion

### 4.1. Validation of the Ellipsoid Fitting Method

Evaluating the CT images of the 3D-printed particles and glass beads (RP1, RP2, GBs) using the proposed method, revealed that the particle length was generally identical to the specifications of the particles. Furthermore, evaluations were not significantly different from the particle size and shape measured by PIA for the natural sand (KS). Therefore, the proposed method can accurately approximate not only the particles with smooth surfaces and simple shapes such as a sphere or an ellipsoid, but also soil particles with roughness surfaces and complex shapes without changing the calculation method. The characteristics of the proposed method are shown below.

The first is that the objective function is defined based on the cross-sectional areas of the particles. The surface of natural soil particles does not represent a smooth surface, as with the glass beads, and present with more or less roughness on the surface. Therefore, the methods such as using tools like calipers to directly measure the size of particles [27,28] may be sensitive to the shape of the particle surface. However, the proposed method fit an ellipsoid based on the cross-sectional areas of the particle. Hence, the proposed method is less affected by the shape change of the particle surface since the cross-sectional area near the particle surface is small compared to the cross-sectional area of the whole particle. This makes it possible to fit particles such as average ellipsoids, even if the particle has a rough surface.

The second is that a fitting ellipsoid can be acquired from the results of multiple cross-sectional area calculations using the non-linear least-squares method. As shown in Figure 5, natural soil particles have complicated shapes, with localized neck or protruding portions. Even if there is a large difference between the particle and the ellipsoid at such a cross-section, the particle is fitted as an ellipsoid by considering the cross-sectional areas at other cross-sections. Therefore, it is expected to fit average ellipsoids while reducing the effect of local shape variations. Although 132 cross-sectional areas were calculated for every 15° of θ and ϕ in this study, 1261 cross-sectional areas were calculated for every 5° of θ and ϕ in a preliminary investigation. However, the fitting results were almost the same even though the computation time increased as the number of evaluated cross-sectional areas increased.

The third is that the particles extracted by image segmentation can be directly evaluated. Previous study was fitting an ellipsoid by converting into a mesh model in STL format [43]. In this method, the relationship between the accuracy of the transformation to the mesh model and the fitting results needs to be investigated. On the other hand, there is no necessity to consider the transformation accuracy of the mesh model since the proposed method directly evaluates the particles extracted by image segmentation.

The proposed method can fit the particle as an ellipsoid and simultaneously evaluate the particle length and direction of the long-, middle-, and short-axes of the particle. In addition, since the coefficients in the general equation for ellipsoids (Equation (1)) can be evaluated directly, it is highly suitable for modeling granular materials in numerical analysis such as DEM. On the other hand, before fitting the particles as ellipsoids, the particles were evaluated using a bounding box to measure the particle length with respect to the X-, Y-, and Z-axes, and particles with particle lengths smaller than 10 voxels were excluded from the fitting. This is because when the particle length is smaller than 10 voxels, the accurate cross-sectional area cannot be calculated due to the lack of voxels to represent the particles, which decreases the approximation accuracy of the fitted ellipsoid. Hence, it is suggested that the proposed method should be applied to the particles with a length larger than 10 voxels. The magnification during CT scanning should be set in accordance with the expected particle length.

### 4.2. Relationship between the Changes in Soil Structure and Strain Due to Shearing

The direct shear experiment showed that the shear stress reached its peak at a D_h_ of ~1.3 mm, followed by a strain softening process. In this study, shearing was stopped, and CT scanning was performed at the initial state (S0) and when D_h_ reached 0.3, 3, 5, and 8 mm (S1 to S4, respectively).

In the initial state (S0), the porosity is generally evenly distributed over the whole sample, and the long-axis of most particles was directed horizontally and the short axis vertically. This was because the experimental box was placed on a horizontal table when it was filled with sand, which may have been an effect of the particle’s long axis being deposited in a mechanically stable direction. As the shear progressed and D_h_ reached 8 mm, changes in the porosity were observed in the range of G3–G6, or ~14.6 mm. This corresponds to 7.1 times the median particle size of $d_{m}$ (2.05 mm) measured by PIA. Oda and Kazama (1998) reported that the width of the shear zone is 7 to 8 times larger than the median grain size by microscopically observing the 2D cross section near the shear zone of specimens after shear experiments using Toyoura sand (D_50_ = 0.206 mm) and Ticno sand (D_50_ = 0.527 mm) [1]. In this study, shear experiments using KS (D_50_ = 2.05 mm) were conducted to evaluate the change in porosity in 3D space, and the width of the shear zone was generally similar. However, it was clarified that the porosity changes slowly as the shear progresses, and that the porosity inside the shear zone increased in variability. Similar to the porosity, a decrease in CSR was observed in the range of G3–G6, indicating a loss of interlocking between particles due to shearing. The volume strain and the shear strain also changed in the range of G3–G6. In particular, the regions where the porosity changed (Figure 12(a1,a2)) and the volumetric strain occurred (Figure 13(a1,a2)) generally correspond to each other, suggesting that the shear changed the porosity and caused the volumetric change. Previous studies have reported that the volume change caused by the progress of shear is largely due to the effect of particle rotation, which occurs when a particle rides up or rides down on a neighboring particle [1]. The relationship between the volume change and particle rotation in the shear zone has been pointed out by numerical simulations using DEM by considering the resistance between particles [44] and particle shape [45]. On the other hand, in this study, the evaluation of soil structure inside the actual shear zone showed that the particles rotated in the range of the volumetric strain, suggesting that the volume change and particle rotation are closely related, similar to the previous study. However, the region where the change in particle direction occurred was narrower than the region where the change in the volume strain occurred.

Immediately after the shear stress exceeded the peak and transitioned to the strain softening process (S2, D_h_ = 3 mm), volume expansion was dominant inside the shear zone. On the other hand, some particles were visually observed to have rotated on the CT image, but there was no significant change in the histogram showing particle direction. This suggests that there were only a few particles that produced rotation that caused volume expansion.

As strain softening progressed (S4, D_h_ = 8 mm), the minimum volumetric strain decreased further, and the maximum volumetric strain also increased further. However, the average volumetric strain approached zero and the state reached equilibrium (or steady state). In contrast, according to the change of particle direction with time, the change of particle direction became sharper after S3 (D_h_ = 5 mm) when the volume expansion inside the shear zone exceeded the peak. Finally, the ratio of horizontally directed long axes of the particles decreased by ~6%, and the ratio of vertically directed short axes of the particles decreased by a maximum of ~8%. In addition, the ratio of vertical long axes of the particles increased by ~4%, as well as the ratio of the horizontal short axes of the particles increasing by ~4%. These changes in the soil structure were not observed in the region away from the shear plane, which is considered to be a characteristic of the soil structure near the shear plane.

## 5. Conclusions

The purpose of this study was to model the shear deformation in granular materials and to quantitatively evaluate the soil structure by image analysis of CT images. Firstly, the ellipsoid fitting method on CT images was proposed. Ellipsoid particles manufactured using a 3D printer, glass beads, and sand were filled into acrylic glass columns, scanned using micro-focus X-ray CT, and the proposed ellipsoid fitting method validated on the CT images. Secondly, the direct shear experiment was conducted, and micro-focus X-ray CT was performed to evaluate the soil structure as it changed with shear deformation. After extracting particles by image segmentation, the ratio of non-particle voxels in the RVE (porosity) and the ratio of voxels corresponding to the contact surface between particles in the RVE (contact surface ratio) were evaluated. The volumetric strain distribution and shear strain distribution were evaluated by DIC analysis. In addition, the proposed method was applied to evaluate the change in particle direction due to shearing. As a result of the above discussion, the following points were clarified.

1. The ellipsoid fitting method proposed in this study is less affected by the irregularities on the particle surface and local shape changes; thus, it can fit particles with complex shapes such as average ellipsoids. The proposed method can accurately fit ellipsoids not only for spherical or ellipsoidal particles produced by glass beads or 3D printers, but also for natural soil particles with surface roughness and complex shapes without changing the calculation method.
2. The specimen of direct shear experiment used in this study was filled by free fall into a shear box placed on a horizontal table, so that the long axis of most of the particles were directed in the horizontal direction and the short axis in the vertical direction in the initial state. Even if shearing occurred, the overall tendency in the direction of the particles is sustained. However, it was clarified that the direction of the particles partially changed when the volume expansion inside the shear zone exceeded the peak. The ratio of both the horizontally directed long axes and vertically directed short axes of the particles decreased by 6~8%. On the other hand, the ratio of both the vertical long axes and horizontal short axes of the particles increased by ~4%. Since no such change was observed in the region away from the shear plane, it was suggested that the change is characteristic of the soil structure near the shear plane.
3. The porosity, contact–surface ratio, volumetric strain, and shear strain changed significantly in the range of ~7.1 times the median grain size of the sand used in this study. On the other hand, it is obvious that the change in particle direction occurs within an even narrower range than the change in porosity, contact–surface ratio, volumetric strain, and shear strain, and is restricted to the vicinity of the shear plane.

Shear zones in soil exist potentially in the initial depositional state and appear with loading, governed by the shape characteristics of the soil particles. The results of this study suggest that the understanding of the sedimentary structure of soil particles in the initial state will enable the determination of the necessary slip lines for soil stability calculations. As X-ray CT systems become more advanced, it is expected that high-resolution images of soil particles will be obtained, which will accelerate research on soil particle behavior. In such situations, an ellipsoid fitting method proposed in this study will be an effective method for understanding the soil particle behavior of the geomaterials.

## Figures and Tables

**Table 1:** Statistical analysis results of ellipsoid fitting.

Material	Parameter	Unit	Mean	Std	Q_25_	Q_50_	Q_75_
RP1	$d_{1}$	[mm]	15.63	0.58	15.67	15.87	15.94
	$d_{2}$		11.72	0.42	11.79	11.85	11.90
	$d_{3}$		3.91	0.13	3.84	3.88	3.90
	$S_{k}$	[-]	0.57	0.015	0.57	0.57	0.57
RP2	$d_{1}$	[mm]	15.52	0.71	15.47	15.84	15.90
	$d_{2}$		7.85	0.24	7.83	7.93	7.97
	$d_{3}$		3.87	0.13	3.82	3.83	3.85
	$S_{k}$	[-]	0.50	0.017	0.49	0.50	0.50
GBs	$d_{1}$	[mm]	0.88	0.14	0.81	0.87	0.96
	$d_{2}$		0.78	0.10	0.74	0.79	0.84
	$d_{3}$		0.76	0.10	0.72	0.76	0.82
	$S_{k}$	[-]	0.92	0.06	0.89	0.94	0.97
KS	$d_{1}$	[mm]	2.55	0.45	2.24	2.48	2.78
	$d_{2}$		1.90	0.22	1.75	1.89	2.05
	$d_{3}$		1.39	0.21	1.26	1.39	1.54
	$d_{m}$		2.02	0.40	1.91	2.05	2.20
	$S_{k}$	[-]	0.75	0.08	0.70	0.75	0.80

## Data Availability

Not applicable.

## Abbreviations

The following abbreviations are used in this manuscript:

CSR	ratio of contact surface between particles on RVE
CT	computed tomography
DEM	distinct element method
D_h_	horizontal displacement of lower box of direct shear experiment’s apparatus
DIC	digital image correlation
GBs	glass beads
KS	Kashima–Keisa sand
PIA	particle image analyzer
RP	resin particles
RVE	representative volume element

## Appendix A

The derivation process for the theoretical formulae of the cross-sectional area of an ellipsoid is summarized as follows. First, the general equation of a two-dimensional ellipse centered at the origin can be expressed as follows:

(A1) $${\mathrm{ax}}^{2}+2\mathrm{hxy}+{\mathrm{by}}^{2}+c=0,$$

(A2) $$\begin{array}{cc} ⟺ & \left[\begin{array}{cc} x & y \end{array}\right]\left[\begin{array}{cc} a & h\\ h & b \end{array}\right]\left[\begin{array}{c} x\\ y \end{array}\right]+c=0,\;\mathrm{and} \end{array}$$

(A3) $$\begin{array}{cc} ⟺ & x^{T}Qx+c=0. \end{array}$$

The area of an ellipse, based on Equation (A1) can be expressed as:

(A4) $$S=\frac{-c\pi }{\sqrt{\lambda_{1}\lambda_{2}}},$$

where S is the area of the ellipse, and $\lambda_{1}$ and $\lambda_{2}$ are the eigenvalues of matrix Q. The eigenvalues were calculated using Equation (A5):

(A5) $$\det\left[\begin{array}{cc} a-\lambda & h\\ h & b-\lambda \end{array}\right]=0\;\mathrm{and}$$

(A6) $$\begin{array}{cc} ⟺ & \lambda^{2}-\left(a+b\right)\lambda +\left(\mathrm{ab}-h^{2}\right)=0. \end{array}$$

Using the formula for the products of the roots of the quadratic equation, as shown in Equation (A6), we can modify Equation (A4) as follows:

(A7) $$S=\frac{-c\pi }{\sqrt{\mathrm{ab}-h^{2}}}.$$

Second, the general equation of a three-dimensional ellipsoid centered at the origin can be expressed as follows:

(A8) $${\mathrm{Ax}}^{2}+{\mathrm{By}}^{2}+{\mathrm{Cz}}^{2}+2\mathrm{Fyz}+2\mathrm{Gzx}+2\mathrm{Hxy}+D=0.$$

The equation of a plane through the origin is given as:

(A9) $$n_{\mathrm{px}}x+n_{\mathrm{py}}y+n_{\mathrm{pz}}z=0\;\mathrm{and}$$

(A10) $$\begin{array}{cc} ⟺ & z=-\left(n_{\mathrm{px}}x+n_{\mathrm{py}}y\right)/n_{\mathrm{pz}}, \end{array}$$

where $n_{\mathrm{px}}$, $n_{\mathrm{py}}$, and $n_{\mathrm{pz}}$ are the normal vectors of the plane.

Equations (A8) and (A10) can be implemented to yield the equation for the cross-sectional area of an ellipsoid along the plane given by Equation (A10). When Equation (A10) is substituted into Equation (A8), we obtain the following:

(A11) $$\begin{array}{c} \left(\frac{{\mathrm{An}}_{\mathrm{pz}}^{2}+{\mathrm{Cn}}_{\mathrm{px}}^{2}-2{\mathrm{Gn}}_{\mathrm{px}}n_{\mathrm{pz}}}{n_{\mathrm{pz}}^{2}}\right)x^{2}\\ \;\;\;\;\;+2\left(\frac{{\mathrm{Cn}}_{\mathrm{px}}n_{\mathrm{py}}-{\mathrm{Fn}}_{\mathrm{px}}n_{\mathrm{pz}}-{\mathrm{Gn}}_{\mathrm{py}}n_{\mathrm{pz}}+{\mathrm{Hn}}_{\mathrm{pz}}^{2}}{n_{\mathrm{pz}}^{2}}\right)\mathrm{xy}\\ \;\;\;\;\;+\left(\frac{{\mathrm{Bn}}_{\mathrm{pz}}^{2}+{\mathrm{Cn}}_{\mathrm{py}}^{2}-2{\mathrm{Fn}}_{\mathrm{py}}n_{\mathrm{pz}}}{n_{\mathrm{pz}}^{2}}\right)y^{2}+D=0. \end{array}$$

The final form of the cross-sectional equation is given by Equations (A12) and (A13), which can be compared with Equations (A1) and (A7).

(A12) $$S_{e}=\frac{-D\pi }{\sqrt{e_{1}+e_{2}+e_{3}+e_{4}+e_{5}+e_{6}}},$$

(A13) $$\begin{array}{cc} \mathrm{where} & e_{1}=\left(\mathrm{BC}-F^{2}\right)n_{\mathrm{px}}^{2},\\ & e_{2}=\left(\mathrm{AC}-G^{2}\right)n_{\mathrm{py}}^{2},\\ & e_{3}=\left(\mathrm{AB}-H^{2}\right)n_{\mathrm{pz}}^{2},\\ & e_{4}=2\left(\mathrm{FG}-\mathrm{CH}\right)n_{\mathrm{px}}n_{\mathrm{py}},\\ & e_{5}=-2\left(\mathrm{BG}-\mathrm{FH}\right)n_{\mathrm{px}}n_{\mathrm{pz}},\\ & e_{6}=-2\left(\mathrm{AF}-\mathrm{GH}\right)n_{\mathrm{py}}n_{\mathrm{pz}}. \end{array}$$

## Appendix B

The calculation methods for the axial radius, rotation axis, and rotation angle from the general equation of a three-dimensional ellipsoid are summarized as follows. The general equation of a three-dimensional ellipsoid centered at the origin can be expressed as follows:

(A14) $${\mathrm{Ax}}^{2}+{\mathrm{By}}^{2}+{\mathrm{Cz}}^{2}+2\mathrm{Fyz}+2\mathrm{Gzx}+2\mathrm{Hxy}+D=0\;\mathrm{and}$$

(A15) $$\begin{array}{cc} ⟺ & x^{T}Qx+D=0, \end{array}$$

(A16) $$\begin{array}{cc} \mathrm{where} & x^{T}={\left[\begin{array}{ccc} x & y & z \end{array}\right]}^{T}\;\mathrm{and} \end{array}$$

(A17) $$Q=\left[\begin{array}{ccc} A & H & G\\ H & B & F\\ G & F & C \end{array}\right].$$

When matrix Q is diagonalized, Equation (A15) can be modified as follows:

$$x^{T}Qx+D=0,$$

(A18) $$\begin{array}{cc} ⟺ & x^{T}P^{T}\left[\begin{array}{ccc} \alpha & 0 & 0\\ 0 & \beta & 0\\ 0 & 0 & \gamma \end{array}\right]Px+D=0, \end{array}$$

(A19) $$\begin{array}{cc} ⟺ & x^{\prime T}\left[\begin{array}{ccc} \alpha & 0 & 0\\ 0 & \beta & 0\\ 0 & 0 & \gamma \end{array}\right]x^{\prime }+D=0,\;\mathrm{and} \end{array}$$

(A20) $$\begin{array}{cc} ⟺ & {\alpha x}^{\prime 2}+{\beta y}^{\prime 2}+{\gamma z}^{\prime 2}=-D, \end{array}$$

(A21) $$\begin{array}{cc} \mathrm{where} & x^{\prime }=\left[\begin{array}{c} x^{\prime }\\ y^{\prime }\\ z^{\prime } \end{array}\right]=P\left[\begin{array}{c} x\\ y\\ z \end{array}\right]. \end{array}$$

Here, α, β, and γ are the eigenvalues of matrix Q and P in the orthogonal matrix.

The equation of an ellipsoid with axial radius directions along the x-, y-, and z-axes can also be expressed as follows:

(A22) $$\frac{x^{2}}{r_{x}^{2}}+\frac{y^{2}}{r_{y}^{2}}+\frac{z^{2}}{r_{z}^{2}}=1,$$

where $r_{x}$, $r_{y}$, and $r_{z}$ are the axial radii of the ellipsoid.

Therefore, the axial radius can be obtained by comparing Equations (A20), and (A22).

(A23) $$r_{x}=\sqrt{-D/\alpha },\;r_{y}=\sqrt{-D/\beta },\;\mathrm{and}\;r_{z}=\sqrt{-D/\gamma }.$$

The rotation axis and angle of an ellipsoid are calculated from the components of the orthogonal matrix [46]:

(A24) $$\theta_{e}=\cos^{-1}\left(\frac{P_{11}+P_{22}+P_{33}-1}{2}\right)\;\mathrm{and}$$

(A25) $$\left[\begin{array}{c} n_{\mathrm{ex}}\\ n_{\mathrm{ey}}\\ n_{\mathrm{ez}} \end{array}\right]=\frac{1}{2\sin\theta_{e}}\left[\begin{array}{c} P_{32}-P_{23}\\ P_{13}-P_{31}\\ P_{21}-P_{12} \end{array}\right],$$

where $\theta_{e}$ is the rotation angle of the ellipsoid, and $n_{\mathrm{ex}}$, $n_{\mathrm{ey}}$, and $n_{\mathrm{ez}}$ are the normal vectors of the rotation axis.
